# Hemoglobin-Based Blood Substitutes and the Treatment of Sickle Cell Disease: More Harm than Help?

**DOI:** 10.3390/biom7010002

**Published:** 2017-01-04

**Authors:** Abdu I. Alayash

**Affiliations:** Laboratory of Biochemistry and Vascular Biology, Center for Biologics Evaluation and Research, Food and Drug Administration, Bethesda, MD 20993, USA; abdu.alayash@fda.hhs.gov; Tel.: +1-240-402-9350

**Keywords:** hemoglobin, blood substitutes, sickle cell disease, heme oxidation

## Abstract

Intense efforts have been made by both industry and academia over the last three decades to produce viable hemoglobin (Hb)-based oxygen carriers (HBOCs), also known as “blood substitutes”. Human trials conducted so far by several manufactures in a variety of clinical indications, including trauma, and elective surgeries have failed and no product has gained the Food and Drug Administration approval for human use. Safety concerns due to frequent incidences of hemodynamic, cardiac events, and even death led to the termination of some of these trials. Several second generation HBOC products that have been chemically and/or genetically modified (or in some cases ligated with carbon monoxide (CO)) found a new clinical application in conditions as complex as sickle cell disease (SCD). By virtue of higher oxygen affinity (P_50_) (R-state), and smaller size, HBOCs may be able to reach the microvasculature unload of oxygen to reverse the cycles of sickling/unsickling of the deoxy-sickle cell Hb (HbS) (T-state), thus preventing vaso-occlusion, a central event in SCD pathophysiology. However, biochemically, it is thought that outside the red blood cell (due to frequent hemolysis), free HbS or infused HBOCs are capable of interfering with a number of oxidative and signaling pathways and may, thus, negate any benefit that HBOCs may provide. This review discusses the advantages and disadvantages of using HBOCs in SCD.

## 1. Introduction

Growing interest in the production of artificial blood over the last three decades has resulted in several products that would have revolutionized the practice of blood transfusion. These products are shelf-stable, portable, “one-type-fits-all” blood substitutes that were designed to replace standard blood transfusions in extreme, life-threatening situations, such as trauma [1]. Although blood transfusion in the United States is considered a safe practice, concerns regarding the acquired immune deficiency syndrome epidemic and human immunodeficiency virus contaminated blood (or other infectious agents) have stimulated both industry, as well as the military in developing products as alternatives to blood donation [2,3].

The development of these products has stalled because of problems with their safety and efficacy. In addition, changes introduced onto the hemoglobin (Hb) molecule by chemical or genetic modifications presented considerable barriers to the full understanding of how these molecules operate in a cell-free environment (for review see [4,5]). Three major biochemical mechanisms were put forward by researchers to explain the basis of free Hb-mediated toxicity that would otherwise been suppressed inside the red blood cell. These are: (1) scavenging of endothelial nitric oxide (NO), a vasodilator; (2) oversupply of oxygen; and (3) heme-mediated oxidative reactions (for review see [6,7]).

Hemodynamic imbalances (as manifested in blood pressure elevation) in response to Hb-based oxygen carriers (HBOC) infusion are viewed by many as a critical step which result from NO scavenging by Hb. While the depletion of NO (an autacoid that acts mainly in the microenvironment of cells) may explain many physiological effects, including vasoconstriction and hypertension (which are limited in duration to 1–2 h in both animals and humans), no tangible long-term consequences have been definitively attributed to this reaction [8]. An alternative mechanistic explanation to NO scavenging is the hypothesis of premature oversupply of oxygen to tissues. This results in an auto regulatory vaso-constriction and/or through the formation of reactive oxygen species and local destruction of NO [2]. Other, less-studied enzymatic activities initiated by endogenous oxidants as they react with the Hb heme moiety may have more lasting tissue-damaging effects than the other two mechanisms and are currently under intense investigation [9,10]. However, the multiplicity of these biochemical hypotheses and conflicting experimental data have complicated the interpretation of many preclinical animal studies that have failed to predict the adverse outcome, observed in the clinical investigations of these therapeutics [11].

## 2. HBOC Types and Indications

Several chemically or genetically engineered HBOCs have been developed with “theoretically” desirable oxygen binding properties, circulatory half-lives and acceptable oncotic properties. The purpose of these chemical/genetic alterations was to primarily serve two functions; first, to stabilize the Hb molecule (which dimerizes readily in dilute solutions) in tetrameric or polymeric form and secondly, to improve Hb oxygen carrying capabilities. The oxygen affinity, as reflected by the P_50_ values (when Hb is half saturated with oxygen) varied among these compounds, ranging from as low as P_50_ = 4.0 mmHg to as high as 40 mmHg. Generally, the following categories of modifications were introduced in most commonly tested HBOCs so far in humans (for review see [7,12]).

### 2.1. Crossed Linked Tetrameric Hemoglobins

Diaspirin-crossed linked Hb (DCLHb) (HemAssist), also commonly known as DBBF Hb, was developed independently by Baxter (Deerfield, IL, USA) and the US Army. DCLHb essentially involved utilizing the reagent (3,5-diobromo-salicyl)-fumarate to crosslink the two alpha subunits (Lys99 α1 and Lys99 α2) of stroma free Hb (SFH) in the deoxy conformation [13]. This HBOC had substantially longer half live than unmodified SFH and a P_50_ value of approximately 30 mmHg, close to that of normal red blood cell. DCLHb had been extensively tested in various preclinical and clinical studies. However, in Phase III studies, patients treated with DCLHb had significantly higher mortality rates than those of the control groups [14]. Somatogen (Boulder, CO, USA) opted for engineering another crosslinked tetramer using recombinant technology (Optro). This Hb was expressed in *Escherichia coli* in the deoxy form of a low oxygen affinity mutant, Hb Presbyterian (βN109K) using glycine to bridge the two α subunits (di- α-gly- α) [15]. Like DCLHb this Hb had a P_50_ value close to that of normal red blood cells (RBCs). Some Phase I/II clinical trials in elective surgeries with Optro (5 g Hb/dL) were conducted but discontinued due to the hypertensive effects and other related adverse events [16].

### 2.2. Conjugated “Decorated” Tetrameric Hemoglobins

Both human and bovine Hbs have been modified by non-protein entities such as polyethylene glycol (PEG) or polyoxyethylene (POE) to primarily increase their retention time in circulation and to maintain low oxygen affinity capabilities. One such HBOC product, pyridoxylated Hb conjugated with POE (PHP), has been tested in humans initially as an oxygen carrier [17]. Apex Inc. (Chapel Hill, NC, USA) later developed this HBOC as a NO scavenger in response to septic shock [18]. In another commercial example, site-specific modification of the thiol surface with imniothiolane, followed by the reaction of the protein with PEG-50 was employed to produce MP4 or Hemospan by Sangart Inc. (San Diego, CA, USA) [19]. This particular Hb unlike other HBOCs had a very high oxygen affinity (P_50_ = 4.0 mmHg) and was introduced specifically to counter the autoregulatory responses associated with first generation HBOCs. MP4 was indicated for use in elective surgery and had gone through early clinical trials in Sweden in orthopedic patients [20]. Sangart failed to secure new funding and had to terminate development operations [6].

### 2.3. Polymerized Human and Bovine Hemoglobins

Intravascular retention times of HBOCs can be further increased by polymerization of the protein. For example, gltutaraldehyde (a 5-carbondialdehyde non-site specific reagent, forms a Schiff-base with Hb lysine amino acid side chains) was used to routinely polymerize human Hb (Polyheme, produced by Northfield (Evanston, IL, USA) and bovine Hb (Hemopure, produced by Biopure Cambridge, MA, USA). Both HBOCs advanced to Phase III clinical trials in humans. Polyheme was initially evaluated in Phase II clinical studies in acute hemorrhage and later in a pivotal Phase III pre-hospital trauma study [21,22]. Hemopure is approved in South Africa for treatment of adult surgical patients who are acutely anemic and for the purpose of eliminating, reducing, or delaying the need for allogeneic red cell transfusion in these patients. Hemopure was tested in the US for a similar indication in orthopedic surgical patients [23,24].

Hemolink is another polymerized Hb with similar oxygen carrying characteristics (P_50_ is around 30–40 mmHg) with non-cooperative behavior (Hill coefficient (*n*) = 1, instead of being equals to 2.5–2.9 for normal human Hb) was produced by a Canadian company, Hemosol Inc. (Toronto, ON, Canada). Hemolink, an intra and inter-molecularly crosslinked Hb with activated sugar, *O*-raffinose has been shown to be unstable, likely due to the none-site specific nature of this form of modification [25]. Hemolink has been tested in a series of clinical trials including in patients undergoing elective coronary artery bypass graft surgery (CABG) [26,27].

## 3. Toxicity of Hemoglobin-Based Oxygen Carriers

The development of HBOCs as viable oxygen therapeutics has been hampered by several safety concerns that have challenged industry, research and regulatory communities. Results of clinical trials and organ-specific aspects of safety were presented in 2008 at a public workshop by industry together with some underlying mechanisms proposed by researchers that could potentially explain these toxicities. Results from this large public presentation of uncensored data were detailed later in a comprehensive publication [28]. Table 1 summarizes some of the adverse events that were commonly reported as result of HBOC infusion. There is also a large body of published preclinical data on the use of some of these HBOCs or their analogues in the literature [29].

Based on preclinical and clinical studies, toxicities associated with HBOCs appear to have a common biochemical origin that emanate from, and are driven by, the heme prosthetic group of Hb [30]. A clear example is the reaction between HBOCs and NO, an important signaling and vasodilator diatomic gas produced by the vascular system. The reaction is primarily with the heme group, which can be completed within a few seconds with a profound consequence, i.e., blood vessel constriction and elevation in systematic and pulmonary blood pressures (approximate mean arterial blood pressure changes ranges between 15 and 30 mmHg) [31]. However, blood pressure elevations seen in animals and in humans appear to follow a predictable path that can return to normal within two hours. Short-term side effects were observed however in animals that can be explained by NO scavenging included transient heart lesions [32], histo-immunological changes in kidneys [33], and gastrointestinal effects in humans [28]. Several strategies that focused on controlling hemodynamic imbalances after infusion of HBOCs using NO donors or enhancing NO synthetic pathways seemed to have blunted these responses, but with little or no long-term tangible improvements on organ toxicities [34]. Efforts to resolve HOBC toxicities have focused primarily on short-term, but long-term consequences similar to those reported for blood transfusion also present serious challenges [35]. Some of the most imaginative and short-term strategies to control blood pressure elevation, including the transformation of the Hb into an NO carrier (*S*-nitrosylation of βCys93 residue) or enzymatically transforming Hb in the presence of nitrite into a source for NO (nitrite reductase) have been advocated [36,37]. But these approaches have also failed to resolve long-term toxicities associated with HBOCs. In fact, infusion of nitrite with an HBOC produced a profound cytotoxicity in the lungs of a swine animal model [38]. Nitrite is known to accelerate Hb oxidation and inducing tissue toxicity [39]. Similar approaches to control pulmonary blood pressure (triggered by free Hb) have resulted in disappointing news from a failed clinical trial that investigated a similar NO modulating strategy in sickle cell disease (SCD) [40].

Recent animal studies showed that Hb compartmentalization (rather than short-lived NO-based therapies) may be useful in countering vasoactive and oxidative toxicities associated with free Hb in hemolytic anemias and Hb oxygen therapeutics [41]. In dogs, guinea pigs, and sickle cell mice models, haptoglobin (Hp) and hemopexin (Hxp) limited the toxic effects of infused cell-free Hb [42]. Additionally, data obtained from these models revealed that Hb–Hp complex formation attenuated the hypertensive response during Hb exposure, and prevented Hb peroxidative toxicity in extravascular compartments, such as the kidney [43]. However, chemical or genetic manipulations of Hb and/or Hp molecules were required to allow effective binding of the two proteins (Hp binds avidly to Hb dimers and only weakly to β-crosslinked HBOCs [44]).

## 4. Sickle Cell Disease

Sickle cell disease affects over 100,000 people in the United States and millions of people worldwide. The disease is caused by a mutation at the β6 position of Hb (β6Glu → Val), which results in the creation of a hydrophobic (sticky) patch (Val6) on the surface of the molecule in a close proximity to another hydrophobic amino acids (Phe85 and Leu88). The polymerization of deoxy-sickle cell Hb (HbS) and subsequent aggregation into long fibers are believed to be the primary molecular events that lead to hemolytic anemia after several cycles of sickling and unsickling of RBCs [45]. An important aspect of the hemolysis is the release of large quantities of Hb in circulation which can contribute to the complications of blood vessel injury and inflammation. It is also well recognized that plasma levels of free Hb that result from hemolysis can be as high as 25 µM during sickle cell crisis, with basal Hb levels at 5–10 µM in sickle cell patients [46].

**Figure 1:**
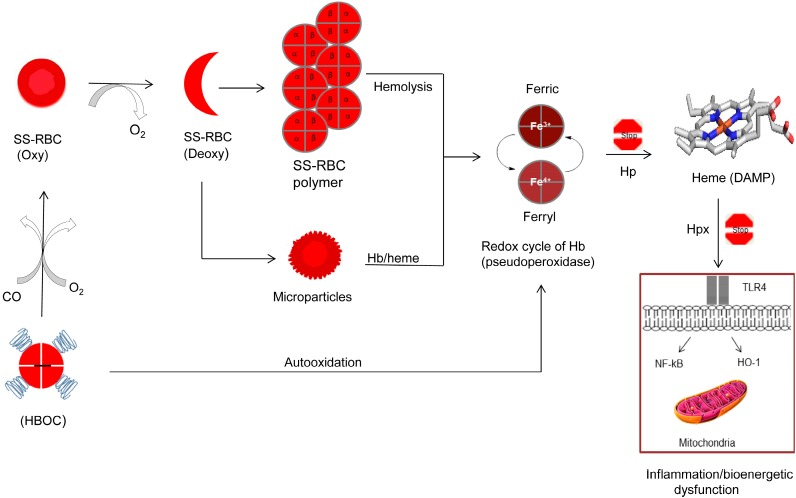
Proposed antisickling functions of hemoglobin-based oxygen carriers (HBOCs) and possible complications of heme oxidative pathways. Red blood cell with predominately sickle cell hemoglobin (HbS) undergoes a vicious sickling and unsickling cycle in the microvasculature resulting in deoxyHbS fibers and free hemoglobin (Hb) spilling out of ruptured cells. Hb-laden microparticles release their contents (Hb and heme). Free Hb undergoes uncontrollable oxidation and oxidative changes through its classic pseudoperoxidase activity (redox cycling between ferric and ferryl heme) resulting ultimately in the loss of heme. Heme as damage associated molecular pattern (DAMP) molecule triggers Toll-like receptor 4 (TLR4), nuclear factor kappa-light-chain-enhancer of activated B cells (NF-kB), and heme oxygenase-1 (HO-1) activation. This leads to inflammatory responses and altered cell metabolism, including mitochondrial dysfunction. Haptoglobin (Hp) (protein scavenger) and hemopexin (Hxp) (heme scavenger) can effectively inhibit Hb oxidative side reactions and heme toxicity. HBOCs due to their relatively smaller sizes can reach microvasculature and by delivering oxygen (O_2_) and/or carbon monoxide (CO) may reverse sickling. However, HBOCs may aggravate oxidative stress by initiating its own damaging oxidative pathways. SS RBCs: sickle cell red blood cells.

In addition, recent reports showed that Hb/heme-laden membrane microparticles (MPs) can also transfer heme to vascular endothelium and mediate oxidative stress, vascular dysfunction, and vaso-occlusion. Heme derived from the hemolysis of sickle cell (SS) RBCs acts as a damage-associated molecular pattern (DAMP) molecule that can activate the Toll-like receptor-4 (TLR4) of the innate immune system leading to oxidant production, inflammation and vascular injury [47] (Figure 1). We have recently shown that free HbS is prone to oxidative changes in presence of oxidants such as peroxide (H_2_O_2_) and that Hb becomes more damaging not only to itself (mainly βCys93 ) but to other biological molecules and organelles such as the mitochondria. This is due in part to HbS’ unique oxidative pathways that result in more persistent oxidizing intermediates, such as the ferryl Hb and its protein radicals [48].

## 5. Do HBOCs Have a Therapeutic Role in Hemoglobinopathies?

Despite years of investigation of the SCD, patients still suffer from hemolysis and blockage of small blood vessels resulting in organ damage and early death. Strategies for sickle cell therapy have been focused on using reagents that directly interfere with the sickling process, by decreasing RBC deoxyHbS concentrations including the induction of fetal Hb and bone marrow transplant [49]. More recent approaches in the treatment of this disease have been focused on reducing inflammation via drugs that target specific inflammatory pathways; these approaches present an attractive alternative therapeutic strategy to ameliorate many of the clinical symptoms of SCD [50].

Hydroxyurea (HU) is the only Food and Drug Administration (FDA) approved drug, which has been shown to be less uniformly effective in patients. FDA approved HU in 1967 originally for the treatment of neoplastic diseases, but it was later approved by the same institution for the use in adults with sickle cell disease. Administered orally (approximately 15–35 mg/kg/day) as a single dose, HU has been shown to boost the levels of fetal Hb (Hb F, α2γ2) in SCD patients. This lowers the concentration of Hb S within the cell resulting in less polymerization of the abnormal Hb [50]. However, the mechanisms by which it increases Hb F are unclear and it has been suggested recently that HU may have other beneficial effects [51].

Following failure to develop a viable HBOC for any of the clinical indications described earlier, new applications in which these products can be clinically useful including potential application to sickle cell disease have been investigated. HBOCs have theoretically several attractive attributes that can be utilized in reversing the sickling process. Among those attributes are smaller molecular sizes, ability to deliver oxygen and reduced reactivity towards NO that can be tailored in HBOCs [52].

## 6. Second-Generation HBOCs and the Treatment of Sickle Cell Anemia

### 6.1. Hemospan (MP4)

Initially MP4 was developed as an oxygen carrier; however, it has recently been re-evaluated as a carbon monoxide (CO) carrier (CO-MP4). Hb ligated to CO undergoes little or no auto-oxidation of its heme group, as long as CO remains ligated to the heme iron. Additionally, CO is now recognized as a cell signaling molecule with cytoprotective and vasodilatory properties [53]. In a rat model of myocardial infarct, in contrast to oxy-MP4, CO-MP4 reduced infarct size when administrated prior to the induction of ischemia [54]. In another experiment, CO-MP4 was found to modulate, heme oxygenase-1 (HO-1), inflammation, and vaso-occlusion in transgenic sickle cell mice. These effects were mediated by nuclear factor E2-related factor 2 (Nrf2), an important transcriptional regulator of HO-1 [55]. However, a planned Phase II study was withdrawn and the sponsor has ceased operations [56].

### 6.2. Sanguinate

Sanguinate is a purified bovine Hb manufactured by Prolong Pharmaceuticals (Piscataway, NJ, USA) and is conjugated with 5000 molecular weight of PEG residues on the surface lysines and is ligated to CO [57]. The unligated form of this HBOC has a high oxygen affinity (a P_50_ of approximately 11 mmHg). In a topload transfusion rat model, PEG-CO (<1 g/dL) produced noticeable reduction in infarct volume [58]. In a Phase I trial, three cohorts of eight healthy volunteers received single ascending doses of Sanguinate (80, 120, or 160 mg/kg, respectively) that were well tolerated. Phase Ib studies have been completed in stable patients with sickle cell disease, but no published data was available in the open literature [59].

### 6.3. Hemopure

A Phase I/II study using polymerized bovine hemoglobin (HBOC-201, Hemopure) was carried out in adult patients with sickle cell disease who were not at a crisis at the time of the study [57]. In this investigation, 18 adults with SCD who were asymptomatic at the time were enrolled for this study. None of the patients who received this product experienced toxicity. There was a significant improvement in heart rate response to identical aerobic exercise workload among those who received HBOC-201 and subjects who received placebo [60]. It is not known whether this indication has been actively pursued by this manufacturer or if another subsidiary is evaluating this product.

### 6.4. HRC 101

HRC 101 has been recently developed by Hemosol Inc., a Canadian manufacturer as a second-generation HBOC for the treatment of sickle cell disease. HRC-101 is a high molecular weight HBOC covalently conjugated with oxidized hydroxyethyl starch. HRC-101 contains less than 10% Hb-hydroxyethyl starch that are less than 192 kDa, the remainder comprising high-molecular-weight Hb-hydroxyethyl starch conjugates. HRC 101 was found to decrease sickle cell related mortality during exposure to acute hypoxic stress in transgenic mice expressing Hb SAD (α_2_^human^ β_2_^S, Antiles, D-Punjab^) [61].

## 7. Do We Need to Be Concerned with Iron Oxidation in Both Sickle Cell and the HBOC Proteins?

### 7.1. Oxidation Reactions of HBOCs

Oxidative toxicity of Hb and the consequences of its redox side reactions are difficult to study in living systems but recent animal studies confirmed the involvement of oxidation reactions in the initiation of inflammatory responses [46,47]. Hb undergoes oxidation, in which the oxygen-bound ferrous (Fe^2+^) heme iron atom spontaneously oxidizes to the ferric/metHb (Fe^3+^) state (autooxidation), initially generating a mixture of protonated and anionic superoxide radicals. Autooxidation of Hb is associated with subsequent globin dysfunction and instability due to the generation of H_2_O_2_ resulting from dismutation of the initial superoxide products [62]. In the presence of excess H_2_O_2_, the pseudoperoxidase catalytic cycle of Hb proceeds with three distinct steps: (1) initial oxidation of HbFe^2+^ to a higher oxidation ferryl Hb (HbFe^4+^); (2) autoreduction of the ferryl intermediate to HbFe^3+^; and (3) reaction of HbFe^3+^(metHb) with an additional H_2_O_2_ molecule to regenerate the ferryl intermediate/ferryl protein radical (·HbFe^4+^=O) (Figure 1). This radical may migrate and further damage the protein, including the irreversible oxidation of βCys93, in the so-called “oxidation hotspot” [48,63]. These internal reactions result in the modification of heme, its attachment to nearby amino acids, and the irreversible oxidation of sulfur-containing amino acid side chains, particularly the highly reactive thiol of βCys93. Oxidation of βCys93 to cysteic acid results in Hb dissociation into dimers, higher auto-oxidation rates, and rapid heme loss [63].

Due to the nature of the chemical or genetic modifications employed in the first-generation HBOCs, auto-oxidation of the heme iron and subsequent oxidative changes had been observed to occur at higher rates than in normal, unmodified Hb. Lowered oxygen affinities due to these modifications have been shown to enhance auto-oxidation rates [64], redox potential [65], and heme loss [66]. This led to extensive efforts by many researchers to design countermeasures that can retard and/or control iron/heme oxidation in HBOCs. This ranged from either adding antioxidants (or reductants) to the HBOC solutions or even crosslinking of some of these antioxidants to the protein [67].

The search for naturally-occurring mutant human Hbs to provide structural and/or conformational clues toward the re-design of oxidatively stable HBOCs has been an active area of research [68]. Genetic engineering of Hbs with reduced NO binding kinetics (because of concerns over hypertensive episodes in human trials) led to some newly designed products [69,70].

Animal species, such as guinea pigs, were used as a model for examining Hb oxidative processes because, similar to humans, they lack the enzymatic ability to produce ascorbate which is a powerful reductant capable of controlling intravascular Hb oxidation [71]. It was demonstrated that in vivo oxidation (autooxidation) after infusion of Oxyglobin (FDA approved HBOCs for use in dogs manufactured by Biopure) can compromise the ability of Hb to carry oxygen, as reflected by the suppression of hypoxia inducible factor (HIF-1α) in kidney tissues for only the first 4–6 h after infusion of HBOCs. This is due to the degradation of HIF subunits which occurs under normoxia, but HIF-1α expression (after the first 4–6 h) signals the onset of hypoxia due to both oxidation of the heme iron of Hb as well as its clearance from circulation [72]. Using this model, it was shown that the induction of renal HO-1 and L-ferritin expressions were accompanied by significant iron deposition after infusion of the same HBOC [73]. In a follow up experiment evidence was presented to show that the transfusion of this Hb in guinea pigs suppresses renal antioxidant enzyme expression at the gene and protein level, possibly through epigenetic alterations involving DNA methylation [33].

A recent case of compassionate use of HBOC-201 was reported with some success in a severely injured Jehovah’s Witness patient, for whom survival was considered unlikely. Severe anemia and cardiac hypoxia were reversed after slow co-infusion of this Hb with ascorbic acid, a powerful reducing agent (1 g twice daily). No vasoactive side effects were associated with the treatment, possibly due to the slow infusion, and the patient survived [74].

### 7.2. Oxidation Reactions of Sickle Cell Hemoglobin

Hebbel et al. were first to document that HbS is oxidatively less stable in vitro than human hemoglobin A (HbA) in particular, upon exposure to heat, oxidants, and mechanical shaking [75]. These oxidation-related mechanisms were believed to contribute to the pathophysiology of the disease. HbS auto-oxidizes at faster rates than HbA in solution [76], has greater affinity than HbA to react with membrane aminophospholipids, and, as result, in conversion to metHb (autooxidation) and the generation of reactive oxygen species (ROS), including superoxide ions (O_2_^●−^), and peroxide H_2_O_2_, resulting in greatly enhanced Hb denaturation and partitioning of the released heme in the membrane bilayer [77]. More recently sickle RBCs were shown to exhibit a unique oxidative environment that includes the presence of up-regulated nicotinamide adenine dinucleotide phosphate (NADPH) oxidase catalytic subunits [78]. NADPH oxidase-derived ROS in sickle RBCs may cause direct oxidative damage to a variety of subcellular structures, reducing deformability and resulting in increased RBC fragility and hemolysis. Moreover, NADPH oxidase activity may deplete the cellular pool of NADPH, thus impairing the ability of the RBC to maintain its antioxidant defenses [78].

An intriguing study on the unique oxidative environment within the SS RBCs was recently published in which a link between accelerated redox reactions associated with HbS and protection against malarial infection was established [79]. Ferryl HbS was found to inhibit actin polymerization in malaria-infected HbS RBCs, thereby preventing the malarial parasites from creating its own actin cytoskeleton within the host cell cytoplasm. Although this mechanism appears to explain how HbS confers protection against malaria, it also serves to show that Hb oxidative reactions, including the formation of ferryl Hb can be detected in spite of the presence of RBC antioxidative enzymes [79].

We have recently reported that the ferric/ferryl redox cycle of HbS is compromised as reflected by the inability of ferryl HbS to revert back to ferric results in oxidative damage and mitochondrial dysfunction in lung epithelial cells. These oxidative pathways may contribute to the vasculopathy in sickle cell disease and can be targeted with antioxidants [49]. These oxidative pathways were also documented in transgenic sickle cell mice; the effects of Hb oxidation products on venules as they undergo stasis (little or no blood flow) were monitored in the microcirculation [47]. Infusion of Hb or heme triggered vaso-occlusion in sickle but not in normal mice. MetHb (but not heme-stabilized cyanometHb) also induced vaso-occlusion, indicating heme liberation is necessary. As a follow-up study, Hb-induced vaso-occlusion was blocked by the metHb-reducing agent methylene blue, Hp and the heme-binding protein Hpx. In this study, it was further shown that free heme (released from Hb) elicited vaso-occlusion (stasis) in transgenic sickle mice by binding to endothelial TLR4. The heme-TLR4 complex activated nuclear factor kappa-light-chain-enhancer of activated B cells (NF-κB) and triggered vaso-occlusion through Weibel-Palade body degranulation and adhesion molecule expression [47].

Blood from sickle cell patients also contains microparticles derived from multiple cellular sources, including the RBCs. Hb-laden microparticles can be a source for both highly oxidized Hb and heme [80]. Due to the unique environment within these particles, oxidative reactions described for free HbS were recently observed in circulating particles from sickle cell patients. When these RBCs were incubated with human vascular endothelial cells, higher oxidation forms of Hb heme loss were markedly increased in MPs generated from sickle cell transgenic mouse and from SCD patients which lead to cellular and subcellular changes including mitochondrial dysfunction [81] (Figure 1).

Based on these observations, it is, therefore, not surprising that heme (derived from sickle RBC and/or from their microparticles due to aging and/or hemolysis) has recently been described as a damage-associated molecular pattern molecule driving inflammation [47]. This is consistent with the early proposed role of Hb oxidation and heme in the disease pathophysiology [77].

## 8. Summary and Conclusions

In spite of years of research and development, it is not known whether an HBOC product can effectively deliver oxygen to the microcirculation, let alone to the sites of the sickling RBC. It has been assumed that HBOCs with smaller molecular size, compared to red blood cells, are expected to transport oxygen to the tissues and sickled cells and could potentially reverse sickling, cellular blockage, and open up the capillaries [82]. Oxygen delivery to these sites may reverse or reduce sickling by increasing the delay time (the time between the start of deoxygenation and polymerization of the deoxyHb) and that would permit cells to escape the narrow capillaries before gelation has begun, therefore, resulting in amelioration of the disease. However, it is unclear how HBOCs ligated or unligated with CO can efficiently deliver oxygen and reverse sickling without themselves being subjected to theirown internal oxidative reactions that can potentially aggravate pathogenesis of the SCD (Figure 1). Based on our understanding of Hb oxidative pathways, the design of an antisickling agent with antioxidant properties may, therefore, present an attractive and alternative strategy to the use of HBOCs. It is quite feasible that drugs targeting βCys93 in sickle cell Hb may be beneficial not only in destabilizing the low oxygen quaternary T-structure, but they can also suppress Hb’s own radical chemistry.

## Figures and Tables

**Table 1:** Hemoglobin-based oxygen carriers /HBOCs) associated adverse events ^1^.

Transient hypertension
Gastrointestinal symptoms
Pancreatic and liver enzyme elevation
Myocardial infarction; cardiac arrhythmias
Renal Injury
Mortality

^1^ Some of the most commonly reported adverse events in human trials with current generation HBOCs (for further details see recent summaries of the clinical data presented by manufactures on these HBOCs) [6,28].
